# Molecular characterization of cryptic species of *Anopheles barbirostris* van der Wulp in China

**DOI:** 10.1186/s13071-014-0592-5

**Published:** 2014-12-16

**Authors:** Yan Wang, Jiannong Xu, Yajun Ma

**Affiliations:** Department of Tropical Infectious Diseases, Second Military Medical University, 800 Xiangyin Rd, Shanghai, 200433 China; Department of Biology, New Mexico State University, PO Box 30001 MSC 3AF, Las Cruces, NM 88003 USA; Haikou Center for Disease Control and Prevention, China CDC Key Laboratory of Surveillance and Early- Warning on Infectious Disease, 20 Yehai Rd, Haikou, Hainan, 571100 China

**Keywords:** *Anopheles barbirostris*, Species complex, China

## Abstract

**Background:**

*Anopheles barbirostris* sensu lato belongs to the Barbirostris subgroup of the subgenus *Anopheles* that is distributed in Southeast Asia. Different molecular forms have been identified based on the rDNA-ITS2 and mtDNA-COI sequences. *Anopheles barbirostris* occurs in China. The species status was uncertain due to the lack of molecular characterization. The present study characterized Chinese *An. barbirostris* using rDNA-ITS2 and mtDNA-COI gene sequences. Two cryptic species were identified.

**Findings:**

DNA was extracted from morphologically identified *An. barbirostris* specimens collected in Yunnan and Hainan from China, the sequences of rDNA-ITS2 and mtDNA-COI regions of 40 individuals were amplified and analyzed. The sequence comparison revealed two cryptic species, corresponding to *An. barbirostris* A1/clade III and A2/clade IV, respectively. The molecular characterization updated the species composition of the *An. barbirostris* complex in China.

**Conclusions:**

This study distinguished two molecular forms in the *An. barbirostris* s. l. in China.

## Background

*Anopheles barbirostris* van der Wulp 1884 is a member species of the Barbirostris subgroup of Subgenus *Anopheles*, Genus *Anopheles. Anopheles barbirostris* is distributed in oriental regions, such as India, Sri Lanka, Thailand, Myanmar, Cambodia, Vietnam, Malaysia, Indonesia and Nepal [1]. *Anopheles barbirostris* sensu lato is a species complex, according to the cytogenetic and molecular characteristics and hybridization results [1-8]. Variations in habitats, resting behaviour and feeding preferences have been reported in the *An. barbirostris* s. l. Some of them have been implicated to be responsible for malaria and filariasis transmission in Southeast Asia [9-11]. Morphologically, it is difficult to distinguish member species one from another [4,5,7]. The sequences of ribosomal DNA second internal transcribed spacer (rDNA-ITS2) and mitochondrial cytochrome DNA oxidase subunit I (mtDNA-COI) exhibit a low level of intraspecific variation and a high level of divergence between species. It is this feature that renders the genes useful as molecular characters for taxonomic classification and phylogenetic inference [12-14]. In the case of *An. barbirostris* complex, molecular analyses of rDNA-ITS2 and mtDNA-COI gene revealed different molecular forms. For example, Saeung *et al.* defined A1, A2, A3, A4, and *An. campestris* [4-7], and Paredes-Esquivel *et al.* identified five clades I, II, III, IV and V [1,8].

In China, *An. barbirostris* s. l. was recorded in provinces of Guangdong, Hainan, Yunnan, Zhejiang, Anhui, Guangxi, Sichuan and Guizhou, but it has not been implicated as a vector for human pathogens [15]. The species status was uncertain due to the lack of molecular characterization. In this study, the rDNA-ITS2 and mtDNA-COI sequences were analyzed for the specimens of *An. barbirostris* s. l. from Yunnan and Hainan China. Two cryptic species were identified.

## Methods

### Mosquito collection and species identification

Wild mosquito adults were captured and sampled from Yunnan and Hainan in China and Myanmar. Detailed information on collection sites is presented in Table 1. The adults were captured using tubes or light traps in livestock rooms. The species of *An. barbirostris* s. l. were identified using morphological keys [15]. The specimens were kept individually in tubes with silica gel and stored at −20°C until DNA extraction was performed.

**Table 1:** **Summary of the mosquito collections in this study**

**Code**	**Sites**	**Date**	**Individual number**
ML	Mengla Yunnan, China	10/2009	15
PR	Puer Yunnan, China	07/2010	11
CM	Chengmai Hainan, China	11/2011	6
XH	Lingshui Hainan, China	06/2012	3
LZ	Lazan, Myanmar	09/2010	5

### rDNA-ITS2 and mtDNA-COI gene amplification and sequencing

The genomic DNA was extracted from individual adults using the Insect Tissue DNA Extraction Kit (Aidlab, China). The rDNA-ITS2 and mtDNA-COI fragments were amplified by PCR. Each PCR (50 μL total volume) mixture contains 1 × PCR buffer (TOYOBO, Japan), 0.2 mM of each dNTP, 1 unit of KOD FX (TOYOBO, Japan), 0.1 μM each of the forward and reverse primers and ~50 ng genomic DNA. The ITS2 region was amplified using primers [13], 5.8S (forward 5′-GAA TGT GAA CTG CAG GAC ACA TG-3′) and 28S (reverse 5′-GGG GTA GTC ACA CAT TAT TTG AGG-3′). The COI gene fragment was amplified by using primers [1], COIBAF (forward 5′-TTG ATT TTT TGG TCA TCC AGA AGT-3′) and COIBAR (reverse 5′-TAG AGC TTA AAT TCA TTG CAC TAA TC-3′). The thermal profile for PCR was 94°C for 2 min, followed by 35 cycles of 98°C for 10s, 45°C for 30s and 68°C for 1 min 30 s (ITS2) or 45 s (COI). A final extension temperature of 68°C was set for 7 min. The PCR products were purified and sequenced in both directions using aforementioned primers. In addition, internal primers BAR1 (5′-GGT GTC ACA TGG TAG ATT AC-3′) and BAR3 (5′-GCG ATC CGA AGT TGA AAG TCA-3′) were used for ITS2 sequencing. The chromatographs of sequences were inspected manually.

### Sequence analysis

The ITS2 and COI sequences of all individuals were aligned by Clustal W [16], respectively. The sequences were deposited in NCBI, the GenBank accession numbers were given in Table 2. The sequences were compared with the available sequences of various species of *An. barbirostris* complex in GenBank. A phylogenetic tree was created using MEGA 5.1 [17]. Substitution models were tested before reconstructing ML trees. Variation between sequences were measured by *p*-distance, implemented by MEGA.

**Table 2:** **The molecular identity of the specimens of** ***Anopheles barbirostris*** **s. l. in this study**

**Species molecular form**	**Sites**	**GenBank accession no.**	
		**rDNA-ITS2**	**mtDNA-COI**
*An.barbirostris* A1/clade III	Mengla Yunnan, China	KJ660985- KJ660999	KM610005-KM610019
	Puer Yunnan, China	KJ661000- KJ661010	KM610020-KM610030
	Chengmai Hainan, China	KJ661011- KJ661014	KM610031-KM610034
	Lingshui Hainan, China	KJ661015,KC878681, KC878682	KM610040-KM610042
	Lazan, Myanmar	KJ661016- KJ661020	KM610035-KM610039
*An.barbirostris* A2/clade IV	Chengmai Hainan, China	KJ661021, KJ661022	KM610043, KM610044

## Findings

### rDNA-ITS2

A total of 40 individuals were used in this study, which were collected in 2009–2012 from Hainan (9 specimens) and Yunnan (26 specimens). The remaining 5 specimens were collected at Lazan, a county on the Myanmar side of the China-Myanmar border. The Chinese side is Naban, Yunnan. The ITS2 sequences of these specimens were amplified by PCR and sequenced. The length was in the range of 1600 bp to 1760 bp. The sequences were compared to the existing sequences of the members of *An. barbirostris* complex in GenBank. The sequence comparison revealed two species, *An. barbirostris* A1/clade III and A2/clade IV. Among 40 specimens, 38 hit to A1 (GenBank accession No. AB331555)/clade III (EU812782) with 99% or 100% identity. The remaining two specimens hit to A2 (AB331551)/clade IV (EU812794) with 99% and 100% identity, therefore classified as A2/clade IV (Table 2). The intraspecific variation (*p*-distance) of rDNA-ITS2 sequences was below 0.001, and the interspecific variation between *An. barbirostris*A1/clade III with A2/clade IV was above 0.201.

**Figure 1:**
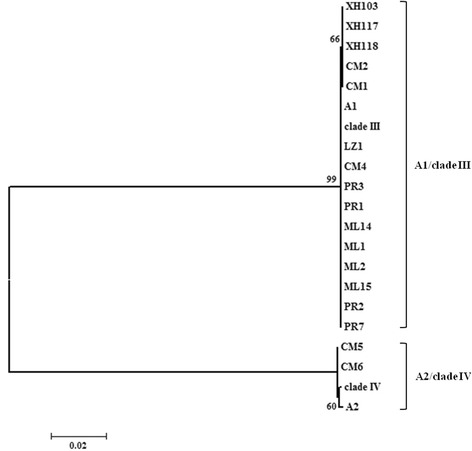
**Maximum likelihood tree created from ITS2 sequences with the GTR substitution model.** The referential sequences were A1 (AB331555) and clade III (EU812782), A2 (AB331551) and clade IV (EU812794). The bootstrap values were shown at nodes.

The relationships of the specimens were further analyzed by maximum likelihood. Different models were tested for making a ML tree. Figure 1 presented a ML tree that was reconstructed by GTR model. Topology of the tree showed two clades, corresponding to *An. barbirostris* A1/clade III and A2/clade IV, respectively. This clearly indicates that there are at least two molecular forms in the *An. barbirostris* complex in China.

### mtDNA-COI

**Figure 2:**
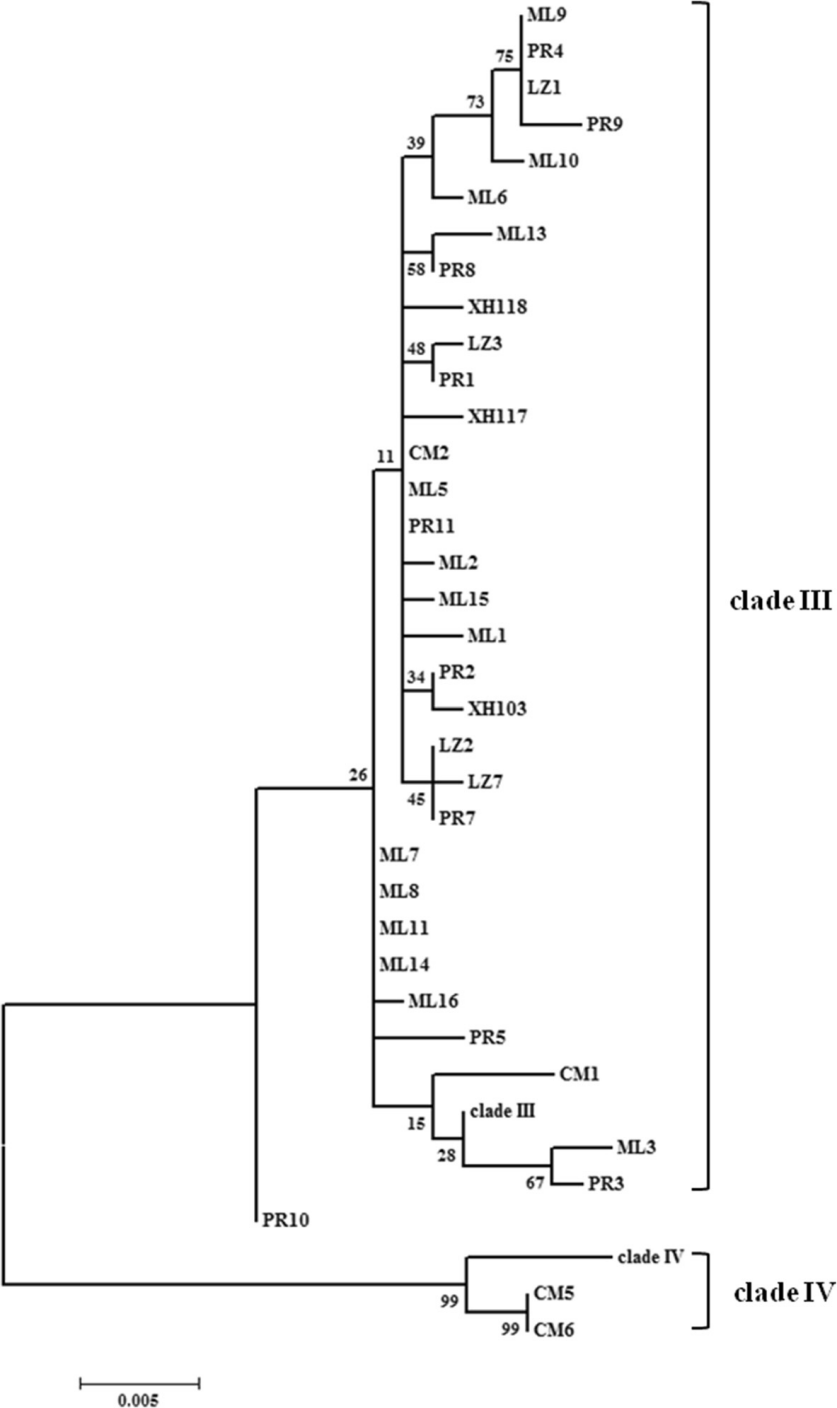
**Maximum likelihood tree created from COI sequences with the HKY + I substitution model.** The referential sequences were clade III (EU797221) and clade IV (EU797257). The bootstrap values are shown at nodes.

A 830 bp fragment of the COI gene was used for the analysis in this study. Out of 40 specimens, 38 sequences were almost identical (99-100% identity) to that of the *An. barbirostris* clade III (EU797221). The remaining two sequences CM5 and CM6 were the same as the *An. barbirostris* clade IV (EU797257). The intraspecific variation (*p*-distance) of mtDNA-COI gene sequences was below 0.012, and interspecific variation between *An. barbirostris* clade III with IV was greater than 0.031. The ML tree using COI sequences was reconstructed by HKY + I model (Figure 2). The specimens were clustered into two clades, corresponding to the *An. barbirostris* clade III and clade IV, respectively. This is consistent with the findings of the ITS2 analysis.

## Conclusions

Both ITS2 and COI are distinctive molecular characters in distinguishing cryptic species in the *An. barbirostris* complex. The sequence comparison identified two molecular forms, A1/clade III and A2/clade IV, in the *An. barbirostris* specimens collected in Hainan and Yunnan. This was the first record documenting the presence of cryptic species in *An. barbirostris* s. l. in China. The molecular characterization will be valuable for phylogenetic study of the genus *Anopheles* in China.
